# Acidophilic haloarchaeal strains are isolated from various solar salts

**DOI:** 10.1186/1746-1448-4-16

**Published:** 2008-10-29

**Authors:** Hiroaki Minegishi, Toru Mizuki, Akinobu Echigo, Tadamasa Fukushima, Masahiro Kamekura, Ron Usami

**Affiliations:** 1 Bio-Nano Electronics Research Center, Toyo University, 2100 Kujirai, Kawagoe, Saitama 350-8585, Japan; 2 Halophiles Research Institute, 677-1 Shimizu, Noda, Chiba 278-0043, Japan

## Abstract

Haloarchaeal strains require high concentrations of NaCl for their growth, with optimum concentrations of 10–30%. They display a wide variety of morphology and physiology including pH range for growth. Many strains grow at neutral to slightly alkaline pH, and some only at alkaline pH. However, no strain has been reported to grow only in acidic pH conditions within the family *Halobacteriaceae*.

In this study, we isolated many halophiles capable of growth in a 20% NaCl medium adjusted to pH 4.5 from 28 commercially available salts. They showed growth at pH 4.0 to 6.5, depending slightly on the magnesium content. The most acidophilic strain MH1-52-1 isolated from an imported solar salt (pH of saturated solution was 9.0) was non-pigmented and extremely halophilic. It was only capable of growing at pH 4.2–4.8 with an optimum at pH 4.4 in a medium with 0.1% magnesium chloride, and at pH 4.0–6.0 (optimum at pH 4.0) in a medium with 5.0% magnesium. The 16S rRNA and DNA-dependent RNA polymerase subunit B' gene sequences demonstrated clearly that the strain MH1-52-1 represents a new genus in the family *Halobacteriaceae*.

## Findings

Halophilic archaea are classified within the order Halobacteriales, family *Halobacteriaceae*, which consists of a number of aerobic extreme halophiles that live in hypersaline environments such as salt lakes, salterns, solar salts, and subsurface salt formation. They require high concentration of NaCl for growth, with optimum concentrations of 10–30%. Currently, the family *Halobacteriaceae *contains 27 genera comprising 95 species that display a wide variety of physiological characteristics including ranges of salinity, temperature, pH etc. for growth. Optimal growth of these haloarchaea has been reported to occur at either neutral or alkaline pH. *Halococcus hamelinensis *100A6^T ^and *Halococcus qingdaonensis *CM5^T ^were reported to be able to grow at pH 4.0–9.0, with an optimum at pH 6.0 [1,2]. However, no strain has been reported to grow only in acidic pH conditions. In this study, we attempted to isolate moderately acidophilic haloarchaea from solar salt samples.

We collected 240 natural sea salts and rock salts available in Japan. Many samples (85) were solar salts, either imported from Australia, France, Mexico, etc. (72 samples), or produced in Japan (13 samples). Fourteen samples were re-crystallized in Japan from the imported solar salts. Fifty three samples were rock salts imported from Bolivia, China, Italy, etc., and 62 were salts produced in Japan from seawater by boiling. Details of the other 26 samples were not clear, since there were no detailed descriptions of the origin on the labels.

We designed a medium MH1 of the following composition (per liter) for enrichment cultures: 4.0 g casamino acids (Difco), 2.0 g yeast extract (Difco), 2.0 g L-glutamic acid, 2.0 g trisodium citrate·H_2_O, 5.0 g K_2_SO_4_, 1.0 g MgCl_2_·6H_2_O, 1.0 g NH_4_Cl, 1.0 g KH_2_PO_4_, 4 mg Fe_2_SO_4_·6H_2_O, 200 g NaCl, 2.0 ml trace metal solution, pH adjusted to 4.5 with 40% KOH and 20 g Bacto-agar (Difco) when necessary. The trace metal solution contained (per liter of distilled water): 2.0 g Na_2_S_2_O_3_·5H_2_O, 1.0 g CaCl_2_·2H_2_O, 0.3 g CoCl_2_·6H_2_O, 0.1 g BaCl_2_·2H_2_O, 0.1 g MnCl_2_·4H_2_O, 0.1 g ZnCl_2_, 0.1 g Na_2_MoO_4_·2H_2_O, 0.1 g NiCl_2_·6H_2_O, 0.04 g AlCl_3_, 0.02 g Na_2_WO_4_·2H_2_O, 0.02 g H_3_BO_4_, pH adjusted to 4.0 with HCl. The medium was autoclaved for 20 min at 121°C.

The salts samples (1.0 g each) were dissolved in 4 ml of MH1 liquid medium. After incubation at 37°C for 1 week without shaking, 0.1 ml of each sample were spread evenly on the MH1 agar plate (pH 4.5) with a spreader. After incubation at 37°C for 2 weeks, colonies were picked, transferred to fresh agar plates of the same pH, and pure cultures were obtained by plating serial dilutions and repeated transfers on the agar plates.

Growth of isolated strains was determined by inoculating pre-cultures of purified strains into test tubes (φ24 × 200 mm) each containing 10 ml liquid media with varying pH (3.0–7.8) and shaken at 37°C with 100 strokes per min. When necessary, growth was monitored by taking 0.1 ml culture broth periodically and measuring absorbance at 600 nm in a 10 mm light path cuvette. After cultivation for 108 hours, 0.1 ml of culture was spread on MH1 agar plates (pH 4.4) and incubated at 37°C for 7 days.

Total DNA was extracted by the method of Cline *et al*. [3]. The 16S rRNA genes were amplified by PCR with the following forward and reverse primers for Archaea: 5'-ATTCCGGTTGATCCTGCCGG and 5'-AGGAGGTGATCCAGCCGCAG. The amplified genes were cloned into pCR2.1 T-vector (Invitrogen) and 6 clones were sequenced using the Big Dye Sequencing Kit Ver. 3.1 (Applied Biosystems) using an ABI 310 DNA sequencer. The 16S rRNA gene sequences retrieved from the DNA Data Bank of Japan [4] were aligned using the CLUSTAL × Multiple Sequence Alignment Program [5]. The phylogenetic tree was reconstructed by the neighbor-joining method [6] and was evaluated by bootstrap sampling [7], expressed as percentages of 1000 replicates.

Twenty eight out of 240 salt samples gave colonies on the first enrichment cultures at pH 4.5. Ten samples were imported solar salts, 6 were rock salts, 2 were salts prepared from imported solar salts (dissolved in seawater and boiled for several hours), and 6 were salts produced in Japan from seawater by boiling. Details of 4 samples were not clear. More than 50 strains were isolated, and they were tested for the ability to grow at pH 4.5–7.0. Most strains grew at pH range of 4.5–6.0, and none of them showed growth at pH higher than 6.5.

**Figure 1:**
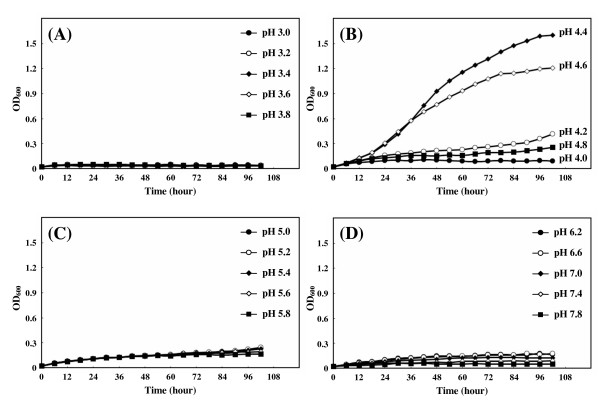
Growth curves (A-D) of strain MH1-52-1 at 37°C in MH1 media adjusted to various pH.

The most acidophilic strain was MH1-52-1 isolated from 'Sango no shio', 'Salt of Coral' in English. According to the manufacture's information (Ochiai.com Co. Japan), the salt is a solar salt imported from Australia, to which powdered coral has been added. The pH of 25% aqueous solution of this salt was 9.0. The strain could grow at pH from 4.2 to 4.8, with optimal growth at pH 4.4–4.6 (Fig. 1B). Almost no growth was obtained at pH lower than 4.0 (Fig. 1A) and higher than pH 5.0 (Fig. 1C–D), demonstrating that the strain MH1-52-1 was moderately acidophilic [8]. The number of surviving cells after cultivation for 108 hours were determined. No colonies were obtained on MH1 agar plates (pH 4.4) from cultures at pH lower than 4.0 and pH higher than 5.8, suggesting that the slight increase in OD660 (Fig. 1C–D may be owing to formation of insoluble materials during shaking at 37°C.

Growth of the strain MH1-52-1 in media with increased MgCl_2_·6H_2_O content was also measured. In media with 50 g per liter, growth was observed at pH 4.0–6.0 with optimum at pH 4.0. The trouble was that crystalline precipitates appeared during cultivation at pH 4.5 or higher, making it impossible to measure the cell density spectrophotometrically. It may be correct to point out that a JCM medium No.168, which supports growth of many haloarchaeal strains, of the following composition (5.0 g casamino acids, 5.0 g yeast extract, 1.0 g sodium glutamate, 3.0 g trisodium citrate·2H_2_O, 20.0 g M_2_SO_4_·7H_2_O, 2.0 g KCl, 36 mg FeCl_2_·4H_2_O, 0.36 mg MnCl_2_·4H_2_O, 200 g NaCl, per liter), but adjusted to pH 4.5 did not support growth of the MH1-52-1. This fact suggests that some components of the medium MH1 (KH_2_PO_4 _or metal solution) is crucial for the growth at acidic pH. The following experiments were thus done in the MH1 medium with 1.0 g MgCl_2_·6H_2_O per liter.

Colonies of the strain MH1-52-1 on 2% agar plate were circular, smooth, non-pigmented after incubation for 7 days at 37°C. Cells were non-motile and pleomorphic, with triangular and disc morphology and lysed in distillated water. The range of NaCl concentration for growth was 10 to 28% (optimum at 20% NaCl), suggesting that this strain was extremely halophilic.

Roughly 500 bp (from 6 to 500, *E*. *coli *numbering) of 16S rRNA gene sequence of 51 strains were determined, and complete sequences of the strain MH1-52-1 and three more representative strains, MH1-16-3 (isolated from a solar salt in Philippines), MH1-34-1 (solar salt from Indonesia), and MH1-136-2 (solar salt in Japan) were determined. The 3 strains could grow at pH 4.5 to 6.0, with optimal growth at pH 5.0–5.5. The 4 sequences differed each other by at least 2%. The sequence of strain MH1-52-1 (AB371717) was most close to that of *Halobacterium noricense *A1^T ^(AJ548827), with 91.7% similarity. A phylogenetic tree reconstructed by NJ method (See additional file 1) suggested that strain MH1-52-1 and the other three strains may represent a novel genus of the family *Halobacteriaceae*. Many isolates (25) clustered very closely with MH1-52-1 as judged from 500 bp of 16S rRNA sequences, while other isolates formed a cluster with MH1-16-3 (18 related isolates) or MH1-34-1 (4 related isolates).

Recently, DNA-dependent RNA polymerase subunit B' gene and protein sequences have been used as an alternative molecular marker in phylogenetic analysis of the family *Halobacteriaceae *[9,10]. Our unpublished data of the sequences of RNA polymerase H, B" and B' genes of all species of the family *Halobacteriaceae *(manuscript in preparation) also suggested that the strain MH1-52-1 was a representative of a novel genus.

According to Johnson [8], there is no common agreement on the pH boundary which delineates acidophily, but a useful guide is that extreme acidophiles have optimum pH for growth of < 3.0 and that moderate acidophiles grow optimally at pH 3–5. Currently, no strain has been reported to grow optimally at pH 3–5 within the family *Halobacteriaceae*. The type strains of *Hcc. hamelinensis *and *Hcc. qingdaonensis*, which were reported to grow at pH 4.0–9.0 [1,2], did not grow at pH 4.5 and 6.0 in our MH1 liquid medium.

Acidic hypersaline lakes are known to be distributed in southern western Australia [11]. They are ephemeral, shallow, have pHs ranging from 1.5 to 4.0, and precipitate halite and/or gypsum in siliciclastic-hosted sediments. Although acidophilic haloarchaea capable of growth at pH 3 [8] have not been isolated, our present data suggest these acidic saline environments may be habitats of these interesting halophiles.

This is the first report on moderately acidophilic haloarchaeon.

## Authors' contributions

HM carried out isolation of strains and characterization, sequencing of 16S rRNA genes and phylogenetic analysis, design of study, and drafted the manuscript. TM did characterization of the isolates, design of study. AE participated in the design of study. TF participated in the design of study. MK participated in the study design and drafted the manuscript. RU participated in the design of study. All authors have read and approved the final manuscript.

## Competing interests

The authors declare that they have no competing interests.

## Supplementary Material

Additional file 1 **Neighbour-joining phylogenetic tree based on 16S rRNA gene sequences showing the relationship between strains MH1-52-1, MH1-16-3, MH1-34-1, MH1-136-2 and type strains of type species of the genera within the family *Halobacteriaceae*.** Two additional species of the genus *Halobacterium *were also added since this was most closely related with the four isolates. Sequences were retrieved from the GenBank database; accession numbers are given in parentheses. Bootstrap values (%) are based on 1000 replicates and are shown for branches with more than 50% bootstrap support. The sequence of *Methanospirillum hungatei *was included as the outgroup. Bar, 0.02 substitutions per site. Click here for file
