# Supplementary figures and images for: Acidophilic haloarchaeal strains are isolated from various solar salts
Source: Saline Syst. 2008 Oct 29;4:16. doi: 10.1186/1746-1448-4-16 (PMC2583988; doi:10.1186/1746-1448-4-16)

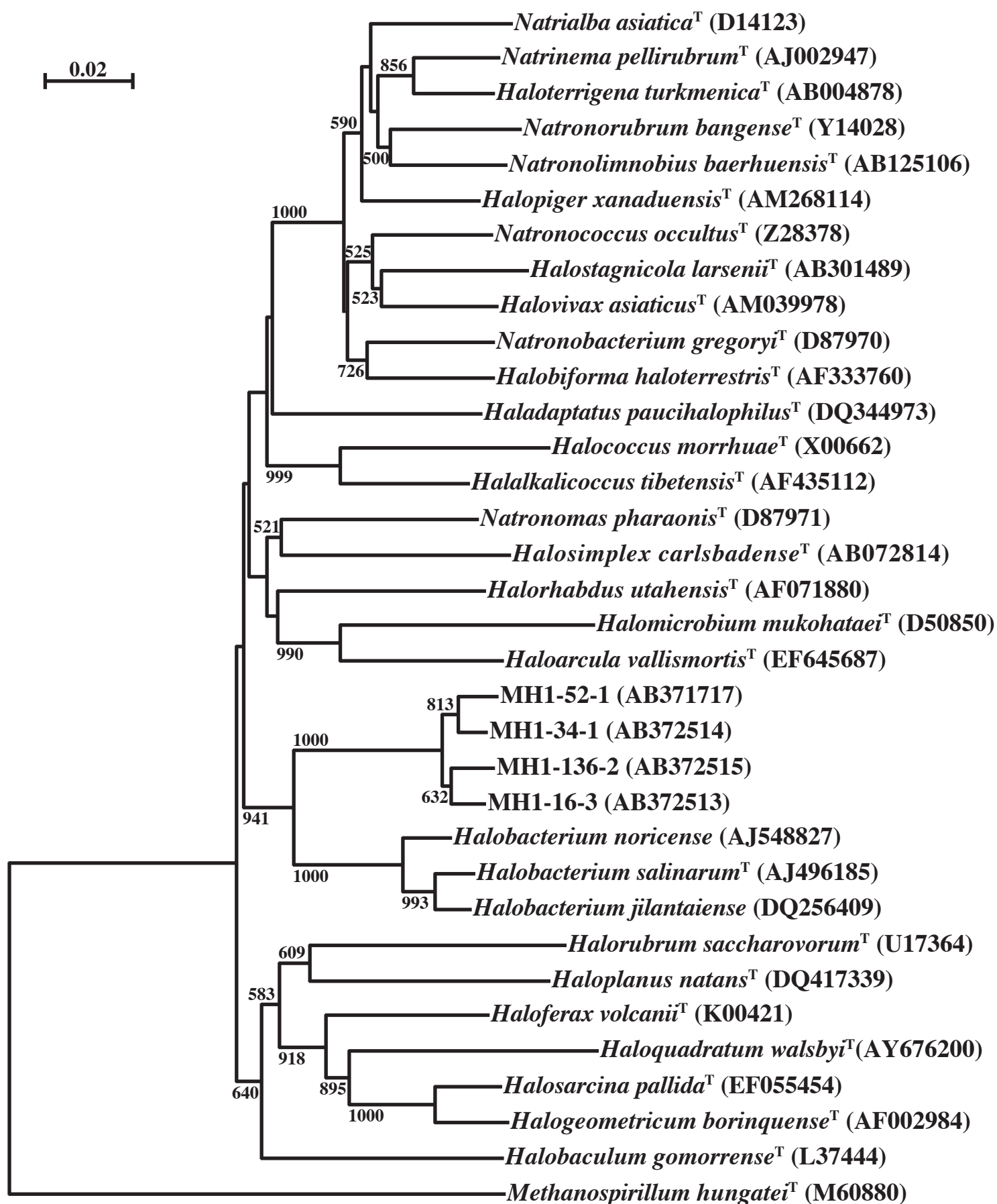

Supplement: Additional file 1 — Neighbour-joining phylogenetic tree based on 16S rRNA gene sequences showing the relationship between strains MH1-52-1, MH1-16-3, MH1-34-1, MH1-136-2 and type strains of type species of the genera within the family Halobacteriaceae. Two additional species of the genus Halobacterium were also added since this was most closely related with the four isolates. Sequences were retrieved from the GenBank database; accession numbers are given in parentheses. Bootstrap values (%) are based on 1000 replicates and are shown for branches with more than 50% bootstrap support. The sequence of Methanospirillum hungatei was included as the outgroup. Bar, 0.02 substitutions per site. [file 1746-1448-4-16-S1.pdf]
